# Host-feeding patterns of *Culex* mosquitoes in Iran

**DOI:** 10.1186/s13071-018-3237-2

**Published:** 2018-12-27

**Authors:** Nariman Shahhosseini, Johannes Friedrich, Seyed Hassan Moosa-Kazemi, Mohammad Mehdi Sedaghat, Mohammad Hassan Kayedi, Egbert Tannich, Jonas Schmidt-Chanasit, Renke Lühken

**Affiliations:** 1 Bernhard Nocht Institute for Tropical Medicine, WHO Collaborating Centre for Arbovirus and Hemorrhagic Fever Reference and Research, Hamburg, Germany; 2 Department of Medical Entomology & Vector Control, Tehran University of Medical Sciences, Tehran, Iran; 3 Department of Parasitology, Lorestan University of Medical Sciences, Khorramabad, Iran; 4 German Centre for Infection Research (DZIF), partner site Hamburg-Lübeck-Borstel-Riems, Hamburg, Germany

**Keywords:** Mosquito, Host species, Blood meal source, Host-feeding pattern, Iran

## Abstract

**Background:**

Different mosquito-borne pathogens are circulating in Iran including Sindbis virus, West Nile virus, filarioid worms and malaria parasites. However, the local transmission cycles of these pathogenic agents are poorly understood, especially because ecological data on vector species are scarce and there is limited knowledge about the host range; this understanding could help to direct species-specific vector control measurements or to prioritize research.

**Methods:**

In the summers of 2015 and 2016, blood-fed mosquitoes were collected at 13 trapping sites on the coast of the Caspian Sea in northern Iran and at an additional trapping site in western Iran. Mosquitoes were generally collected with either a Biogents Sentinel trap or a Heavy Duty Encephalitis Vector Survey trap installed outside. A handheld aspirator was used at the trapping site in western Iran, in addition to a few samplings around the other trapping sites. On average, eight trapping periods were conducted per trapping site. The sources of blood meals were identified using a DNA barcoding approach targeting the cytochrome *b* or *16S* rRNA gene fragment.

**Results:**

The source of blood meals for 580 blood-fed mosquito specimens of 20 different taxa were determined, resulting in the identification of 13 different host species (9 mammals including humans, 3 birds and 1 reptile), whereby no mixed blood meals were detected. Five mosquito species represented more than 85.8% of all collected blood-fed specimens: *Culex pipiens pipiens* form *pipiens* (305 specimens, 55.7% of all mosquito specimens), *Cx. theileri* (60, 10.9%), *Cx. sitiens* (51, 9.3%), *Cx. perexiguus* (29, 5.3%) and *Anopheles superpictus* (25, 4.6%). The most commonly detected hosts of the four most abundant mosquito species were humans (*Homo sapiens*; 224 mosquito specimens, 40.9% of all mosquito specimens), cattle (*Bos taurus*; 171, 31.2%) and ducks (*Anas* spp.; 75, 13.7%). These four mosquito species had similar host-feeding patterns. The only exceptions were a relatively high proportion of birds for *Cx. pipiens pipiens* f. *pipiens* (23.2% of detected blood meal sources) and a high proportion of non-human mammals for *Cx. theileri* (73.4%). Trapping month, surrounding area, or trapping method had no statistically significant impact on the observed host-feeding patterns of *Cx. pipiens pipiens* f. *pipiens*.

**Conclusions:**

Due to the diverse and overlapping host-feeding patterns, several mosquito species must be considered as potential enzootic and bridge vectors for diverse mosquito-borne pathogens in Iran. Most species can potentially transmit pathogens between mammals as well as between mammals and birds, which might be the result of a similar host selection or a high dependence on the host availability.

**Electronic supplementary material:**

The online version of this article (10.1186/s13071-018-3237-2) contains supplementary material, which is available to authorized users.

## Background

Different mosquito-borne pathogens are circulating in Iran, including Sindbis virus (SINV) [1], West Nile virus (WNV) [2] and filarioid worms [3]. The country is under significant risk for the introduction of highly vector-competent, exotic mosquito species and associated pathogens from neighboring countries or through international travel as demonstrated by the recent detection of the invasive Asian tiger mosquito (*Aedes albopictus*) [4] and associated emergence of dengue virus [5].

Information on the hosts of mosquitoes is necessary to identify potential vector species under field conditions [6, 7], so that species-specific control measurements can be directed [8]. There are essentially three types of study method to identify the blood meal source of wild mosquitoes: direct observation of the blood foraging on the host, host-baited traps, and the analysis of the blood content in the mosquito gut [9]. Several studies have analyzed the blood meals of various mosquito species [10]. Before 1996 [11], these studies were mostly limited to a distinction of broad host groups, e.g. “bird” instead of “European blackbird (*Turdus merula*)” [9]. Since the advent of molecular methods, this field of vector research advanced from basic immunological analysis to deoxyribonucleic acid (DNA) barcoding and DNA fingerprinting [9, 12]. In addition, as highlighted by Gunathilaka et al. [13], these polymerase chain reaction (PCR)-based assays are less costly and time-consuming while having a higher sensitivity. Furthermore, cloning of the PCR amplicons might even allow the detection of mixed blood meals.

The mosquito fauna of Iran is diverse, including 64 species and three subspecies in seven genera [14]. However, only a few studies have determined the hosts of mosquitoes in Iran, which have predominantly focused on selected species of the 28 known *Anopheles* species in the country. Basseri et al. [15] and Yeryan et al. [16] examined the feeding patterns of different *Anopheles* species by enzyme-linked immunosorbent assay (ELISA) tests to identify blood meals of human origin. Less than 40% of all specimens had fed on humans. However, studies on other mosquito genera are missing, e.g. *Culex* species, which are the most important vectors of SINV and WNV [1, 2]. In addition, due to the limitations of the previously applied screening techniques, other host-species remain undetermined. DNA barcoding assays give a specific understanding of the selected hosts compared to ELISA assays [9, 12], allowing the closing of the research gap regarding the host-associations of Iranian mosquito species, and thus providing a deeper understanding of the transmission cycles and dynamics of mosquito-borne pathogens [17]. In addition, studies that have estimated the prevalence of mosquito-borne pathogens in Iran were mostly based on the screening of humans [2] or animals [9, 18], whereas only a few studies conducted vector surveillance [19]. Insights into the host range of mosquito species can lead to a prioritized research agenda focused on these species, which may be potential vectors for pathogens [20]. Therefore, with the aim to identify potential mosquito vector species, this study utilized a DNA barcoding approach to identify the hosts of Iranian mosquitoes and analyze the variability of host-feeding patterns in space and time.

## Methods

**Fig. 1:**
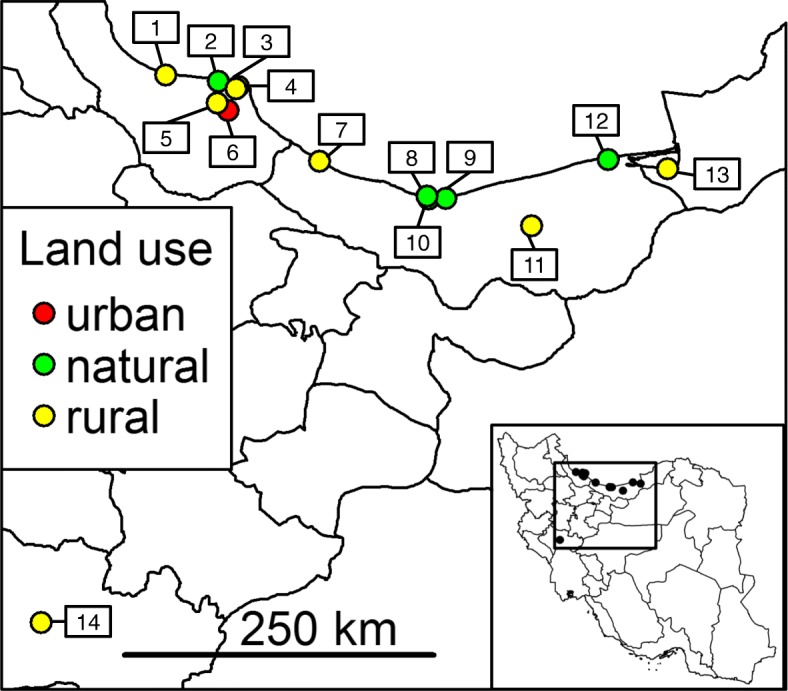
Trapping sites of the analysed blood-fed mosquitoes in Iran with information on the land use

Blood-fed mosquito females were collected in 2015 and 2016 within a study on the distribution of mosquito-borne pathogens at 13 trapping sites in northern Iran along the coast of the Caspian Sea and at an additional trapping site in western Iran [21] (Fig. 1). The surrounding areas around the trapping sites were predominantly characterised as “urban” (1 site), “rural” (8 sites) or “natural” (5 sites). The trapping sites were selected to be representative for the coastal area of the Caspian Sea and protected against vandalism. The sites were characterised by a mixture of natural vegetation (e.g. shrubs, herbs, a few trees), small water bodies (e.g. ditches) and pastures. As the collections were predominantly made on public lands, no specific permissions were required to access the study sites. Permission of each house owner was obtained for indoor samplings. Mosquito collections were conducted with Biogents Sentinel traps (BG trap; Biogents, Regensburg, Germany), Heavy Duty Encephalitis Vector Survey traps (EVS trap; BioQuip Products, Rancho Dominguez, CA, USA), handheld aspirators, and CDC (Centers for Disease Control and Prevention) gravid trap model 1712 (John W. Hook Company, Gainesville, FL, USA) [22]. Due to availability problems, CO_2_ from a gas cylinders for the BG traps was only available in the year 2015 (193 specimens collected with this method). Thus, sugar-fermenting yeast was used in 2016 (Turbo Yeast Pure 48, Alcotec, Dronfield, UK; 5 g yeast, 5 g sugar, 500 ml water) as an organic source of carbon dioxide (85 mosquito specimens). EVS traps with CO_2_ from dry ice collected 27 mosquito specimens and 265 mosquito specimens were collected with handheld aspirators. No blood-fed specimens were collected with gravid traps with a hay infusion as oviposition attractant. BG traps, EVS traps and gravid traps were installed between 7:00 h and 11:00 h, with mosquitoes collected after approximately 24 or 48 h. Due to large distances between the trapping sites, sampling was conducted on different dates. Exact trapping methods per date and sites are provided in Additional file 1: Table S1. In general, one mosquito trap (BG trap or EVS trap) was installed per trapping site. Depending on the availability of dry ice, either EVS or BG traps were used; in a few exceptional cases, both traps were used in parallel. In addition, the handheld aspirator was used in parallel at some trapping dates for the collection of mosquitoes from vegetation around the trapping sites. Between 1 and 36 trapping periods were conducted per trapping site over the two years (on average 7.9 trapping periods).

Sampling with handheld aspirators was conducted in the morning (8:00–11:00 h) or evening (16:00–18:00 h) for 1 h indoors (human dwellings and animal shelters with host nearby) at the trapping site in western Iran (which was not sampled with the other trap types), and 2 h at the other trapping sites to account for the diversity of the outdoor environments. Vegetation or walls of dwellings and shelters were visually checked for mosquitoes and detected specimens sampled with the aspirator.

After collection, mosquito specimens were directly stored on dry ice and kept frozen during morphological identification on chill tables [23, 24]. Furthermore, morphologically identified *Culex pipiens* (*s.l.*) specimens were typed to species level (*Cx. pipiens pipiens* f. *pipiens*, *Cx. pipiens pipiens* f. *molestus* or *Cx. pipiens* cf. *quinquefasciatus*) using a molecular assay [25].

For DNA isolation, the whole body of each mosquito specimen was transferred to a 2 ml tube with 20 pieces of 2.0 mm zirconia beads (BioSpec Products, Bartlesville, USA) and 1 ml of cell culture medium (high-glucose Dulbecco’s modified Eagle’s medium; Sigma-Aldrich, St. Louis, MO, USA). Specimens were homogenised with a Tissuelyser LT (Qiagen, Hilden, Germany) for 2 min at 50 oscillations/s. DNA was extracted from 200 μl of the homogenate using the KingFisher™ Flex Magnetic Particle Processor with the MagMAX™ Pathogen ribonucleic acid/DNA Kit (both Thermo Fisher Scientific, Waltham, MA USA). A polymerase chain reaction (PCR) targeting the mitochondrial cytochrome *b* gene was conducted with a primer pair first published by Kitano et al. [26]: L2513 (5'-GCC TGT TTA CCA AAA ACA TCA C-3') and H2714 (5'-CTC CAT AGG GTC TTC TCG TCT T-3') (~244 bp). However, it is well known that the different primer sets for host-species identification have a high variability regarding their taxa-specific sensitivity [27]. Therefore, if the PCR based on the first primer pair did not work, an additional primer pair targeting the *16S* rDNA fragment was applied in an additional PCR: L14841 (5'-CCA TCC AAC ATC TCA GCA TGA TGA AA-3') and H15149 (5'-CCC TCA GAA TGA TAT TTG TCC TCA-3') (~358 bp) [28]. For each PCR reaction, HotStarTaq Plus Master Mix Kit (Qiagen, Valencia, CA, USA) was used with the following temperature profile for each PCR reaction: incubation at 95 °C for 5 min; 40 cycles at 94 °C for 30 s, 57 °C for 30 s and 72 °C for 30 s; and finally completed by incubation at 72 °C for 5 min. Visualization of amplicons was conducted by electrophoresis in a 2% agarose gel with added Midori Green Advance (Biozym Biotech, Hessisch Oldendorf, Germany). In addition, a positive control (blood from humans, *Homo sapiens*; moose, *Alces alces*; or European blackbird, *Turdus merula*) and negative control (distilled water; Ampuwa, Fresenius Kabi Deutschland GmbH, Bad Homburg, Germany) were used in each PCR. All amplicons were further processed with Sanger sequencing (LGC Genomics, Berlin, Germany), pre-processed with Geneious® 7.1.9 [29] and finally compared to GenBank sequences (http://blast.ncbi.nlm.nih.gov/Blast.cgi).

The statistical computer program R [30] was used for all data analysis. Data manipulation and visualization was conducted with functions from the packages *plyr* [31] and *ggplot2* [32]. Spearman’s rank correlation was used to analyse the statistical relationship between the number of analysed specimens per mosquito species and number of detected host species. Furthermore, only for most abundant mosquito species (*Cx. pipiens pipiens* f. *pipiens*), the statistical influence of the trapping methods (BG trap and aspirator), trapping period (early: June, July; and late: August, September, October), surrounding area (rural, natural) and year (2015, 2016) on the proportion of bird-, human- or non-human mammal-fed mosquito specimens was conducted, using a generalized linear model for each of the three host groups. A Gaussian data distribution and identity link was applied. The full model with the variables trapping method, trapping period, surrounding area, and year was tested using backward elimination of variables based on the Akaike information criterion (AIC) with the R function *step*. The data for the EVS trap were excluded because only four specimens of *Cx. pipiens pipiens* f. *pipiens* were collected with this method. Additionally, the data from the single site with urban surrounding area were removed for this analysis.

## Results

**Fig. 2:**
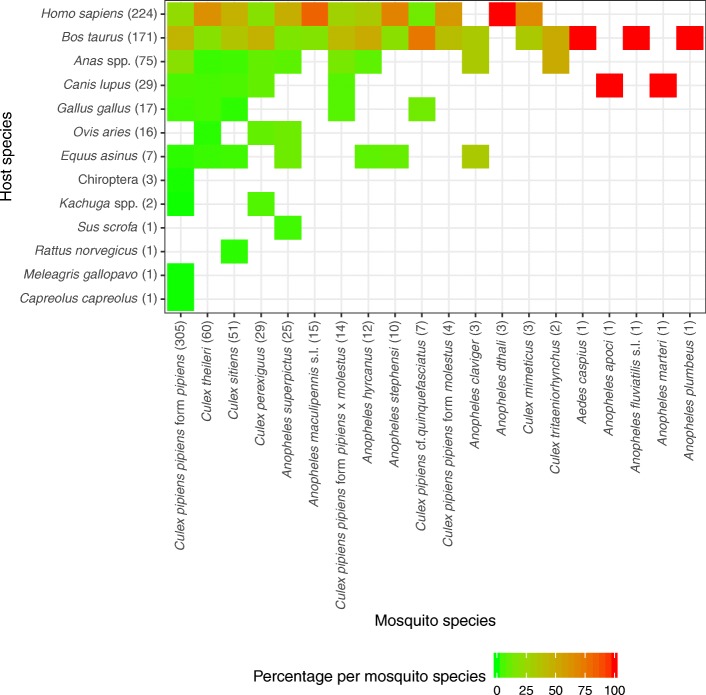
Percentage of each detected host species per mosquito species with the corresponding number of specimens per mosquito species and host species indicated in parentheses

A total of 32,317 mosquito specimens of 28 taxa were collected in 16 trapping sites. All blood-fed specimens were selected and analysed. The source of blood meals for 570 blood-fed mosquito specimens of 20 different taxa was determined, resulting in the identification of 13 different sources of blood meals (9 mammals including humans, 3 birds and 1 reptile) (Fig. 2, Tables 1 and 2, Additional file 1: Table S1). On average, 25.1% of the specimens per sample collected with the aspirator were blood-fed. This proportion was lower for the BG trap (14.7%) and EVS trap (0.5%) (Additional file 2: Table S2). The BG-trap was more efficient in catching *Cx. pipiens* (*s.l.*), while the EVS trap was more efficient in catching *Cx. sitiens* and the aspirator more efficient in catching *Cx. theileri* (Additional file 3: Table S3). No blood-fed specimens were trapped with the gravid trap. Mosquito specimens were predominantly caught in July (189 specimens), August (205 specimens) and September (106 specimens), while a few samples were also collected in June (48 specimens) and October (22 specimens). Of all blood-fed mosquitoes caught, five mosquito species accounted for more than 85.8% of all collected specimens: *Cx. pipiens pipiens* f. *pipiens* (305 specimens, 55.7% of all mosquito specimens), *Cx. theileri* (60, 10.9%), *Cx. sitiens* (51, 9.3%), *Cx. perexiguus* (29, 5.3%) and *Anopheles superpictus* (25, 4.6%) (Table 1). The other mosquito taxa were much rarer and represented by one to 15 specimens. As expected, the total number of detected sources of blood meals had a statistically positive correlation with the number of analyzed specimens (*r*_S_ = 0.93, *P* < 0.001): *Cx. pipiens pipiens* f. *pipiens* (305 specimens, 10 host species), followed by *Cx. sitiens* and *Cx. theileri* (60 and 51 specimens, respectively, each with 7 host species) and *Cx. perexiguus* and *An. superpictus* (29 and 25 specimens, respectively, each with 6 host species).

**Table 1:** Frequency and percentage of each mosquito species with information on the frequency/percentage of detected host-feeding groups (birds, non-human mammals, humans and reptiles) and number of detected host species

Mosquito species	No. (%) of blood-fed mosquito specimens	No. (%) of blood meals taken on humans	No. (%) of blood meals taken on non-human mammals	No. (%) of blood meals taken on birds	No. (%) of blood meals taken on reptiles	No. of host groups	No. of host species
*Culex pipiens pipiens* f. *pipiens*	305 (55.7)	143 (46.8)	87 (28.5)	73 (23.9)	2 (0)	4	10
*Culex theileri*	60 (10.9)	12 (3.9)	43 (14)	5 (1.6)	0 (0)	3	7
*Culex sitiens*	51 (9.3)	19 (6.2)	29 (9.5)	3 (0.9)	0 (0)	3	7
*Culex perexiguus*	29 (5.3)	15 (4.9)	11 (3.6)	3 (0.9)	0 (0)	3	6
*Anopheles superpictus*	25 (4.6)	4 (1.3)	19 (6.2)	2 (0.6)	0 (0)	3	6
*Anopheles maculipennis* (*s.l.*)	15 (2.7)	3 (0.9)	12 (3.9)	0 (0)	0 (0)	2	2
*Culex pipiens pipiens* form *pipiens* × *molestus*	14 (2.6)	6 (1.9)	5 (1.6)	3 (0.9)	0 (0)	3	5
*Anopheles hyrcanus*	12 (2.2)	6 (1.9)	5 (1.6)	1 (0.3)	0 (0)	3	4
*Anopheles stephensi*	10 (1.8)	2 (0.6)	8 (2.6)	0 (0)	0 (0)	2	3
*Culex pipiens* cf. *quinquefasciatus*	7 (1.3)	6 (1.9)	0 (0)	1 (0.3)	0 (0)	2	2
*Culex pipiens pipiens* f. *molestus*	4 (0.7)	2 (0.6)	2 (0.6)	0 (0)	0 (0)	2	2
*Anopheles claviger*	3 (0.5)	1 (0.3)	1 (0.3)	1 (0.3)	0 (0)	3	3
*Anopheles dthali*	3 (0.5)	0 (0)	3 (0.9)	0 (0)	0 (0)	1	1
*Culex mimeticus*	3 (0.5)	1 (0.3)	2 (0.6)	0 (0)	0 (0)	2	2
*Culex tritaeniorhynchus*	2 (0.4)	1 (0.3)	0 (0)	1 (0.3)	0 (0)	2	2
*Aedes caspius*	1 (0.2)	1 (0.3)	0 (0)	0 (0)	0 (0)	1	1
*Anopheles apoci*	1 (0.2)	0 (0)	1 (0.3)	0 (0)	0 (0)	1	1
*Anopheles fluviatilis* (*s.l.*)	1 (0.2)	1 (0.3)	0 (0)	0 (0)	0 (0)	1	1
*Anopheles marteri*	1 (0.2)	0 (0)	1 (0.3)	0 (0)	0 (0)	1	1
*Anopheles plumbeus*	1 (0.2)	1 (0.3)	0 (0)	0 (0)	0 (0)	1	1

**Table 2:** Frequency and percentage for each host species differentiated for four host-feeding groups (birds, non-human mammals, humans and reptiles) of the four most abundant blood-fed mosquito species and over all mosquito species

Host species	*Culex pipiens pipiens* f. *pipiens*		*Culex theileri*		*Culex sitiens*		*Culex perexiguus*		All species	
	*n*	%	*n*	%	*n*	%	*n*	%	*n*	%
Birds										
*Anas* spp.	61	19.4	2	3.1	2	3.8	3	9.4	75	13.7
*Gallus gallus*	11	3.5	3	4.7	1	1.9	0	0	17	3.1
*Meleagris gallopavo*	1	0.3	0	0	0	0	0	0	1	0.2
Total no. of birds	73	23.2	5	7.8	3	5.7	3	9.4	93	17.0
Humans										
*Homo sapiens*	143	45.5	12	18.8	19	35.8	15	46.9	224	40.9
Non-human mammals										
*Bos taurus*	71	22.6	41	64.1	25	47.2	6	18.8	171	31.2
*Canis lupus*	17	5.4	3	4.7	3	5.7	3	9.4	29	5.3
*Capreolus capreolus*	1	0.3	0	0	0	0	0	0	1	0.2
*Equus asinus*	0	0	1	1.6	0	0	3	9.4	7	1.3
Chiroptera	1	0.3	0	0	0	0	2	6.3	3	0.5
*Ovis aries*	6	1.9	2	3.1	2	3.8	0	0	16	2.9
*Rattus norvegicus*	0	0	0	0	1	1.9	0	0	1	0.2
*Sus scrofa*	0	0	0	0	0	0	0	0	1	0.2
Total no. of non-human mammals	96	30.6	47	73.4	31	58.5	14	43.8	229	41.8
Reptiles										
*Kachuga* spp.	2	0.6	0	0	0	0	0	0	2	0.4
Total	314		64		53		32		548	

*Abbreviation*: *n* number of specimens

**Fig. 3:**
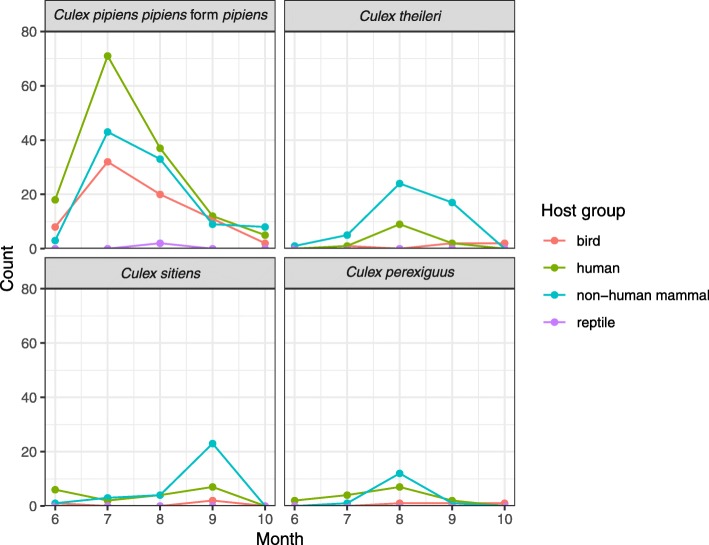
Number of detected specimens per host group for the four most abundant mosquito species differentiated for the trapping month, summed over the two sampling years (2015–2016)

Hosts from three or four host groups were determined for eight mosquito species (40.0% of all species, Table 1). For the four most abundant species, non-human mammals (229 specimens, 41.8% of all mosquito specimens) and humans (224, 40.9%) were the most commonly detected blood meal source, followed by birds (93, 17.0%) and reptiles (2, 0.4%) (Table 2, Fig. 2, Additional file 4: Table S4). The most common non-human mammals were cattle (*Bos taurus*, 171 specimens), dogs (*Canis lupus*, 29 specimens), and sheep (*Ovis aries*, 16 specimens), followed by five species with less than ten detections each (*Equus asinus*, Chiroptera, *Capreolus capreolus*, *Rattus norvegicus*, *Sus scrofa*). With more than 70 records each, humans (*Homo sapiens*; 224 mosquito specimens, 40.9% of all mosquito specimens; detected in 85% of all mosquito species), cattle (171, 31.2%, 60%) and ducks (*Anas* spp.; 75, 13.7%, 45%) were the most common blood meal sources. The four most abundant mosquito species had similar host-feeding patterns, except a relatively high proportion of birds for *Cx. pipiens pipiens* f. *pipiens* (23.2% of detected blood meal sources) and a high proportion of non-human mammals for *Cx. theileri* (73.4%). In addition, 11 mosquito species (55%) fed on both, humans and cattle. No mixed blood meals were detected. The relative proportion of the host-groups did not considerably change in the course of the year for the four most abundant species (Fig. 3, Additional file 5: Figure S1). There were apparent differences between the host-feeding patterns between the three trapping methods (Table 3, Additional file 6: Table S5). For example, the proportion of human-fed mosquitoes were higher for the BG trap compared with the aspirator for *Cx. pipiens pipiens* f. *pipiens* and *Cx. perexiguus*. In contrast, a high proportion of non-human mammal blood sources were found for the aspirator for the four most abundant mosquito species, but it has to be kept in mind that the aspirator was also used more frequently in the rural areas (Additional file 1: Table S1). However, for *Cx. pipiens pipiens* f. *pipiens*, trapping period, trapping method, surrounding area, or year had no statistically significant impact on the proportion of the host groups humans, non-human mammals or birds, i.e. the null model without any variables had the lowest AIC value.

**Table 3:** Frequency and percentage for each host-feeding group (birds, non-human mammals, humans and reptiles) for the four most abundant blood-fed mosquito species differentiated for the three used trapping methods

Mosquito species	Host group	Aspirator (*n* = 55)	BG trap (*n* = 60)	EVS trap (*n* = 30)
*Culex perexiguus*	Bird	0 (0)	2 (13.3)	1 (100)
	Human	5 (31.3)	10 (66.7)	0 (0)
	Non-human mammal	11 (68.8)	3 (20.0)	0 (0)
	Reptile	0 (0)	0 (0)	0 (0)
	Total	16	15	1
*Culex pipiens pipiens* f. *pipiens*	Bird	13 (15.3)	58 (25.8)	2 (50.0)
	Human	31 (36.5)	110 (48.9)	2 (50.0)
	Non-human mammal	40 (47.1)	56 (24.9)	0 (0)
	Reptile	1 (1.2)	1 (0.4)	0 (0)
	Total	85	225	4
*Culex sitiens*	Bird	2 (4.7)	0 (0)	1 (10.0)
	Human	14 (32.6)	0 (0)	5 (50.0)
	Non-human mammal	27 (62.8)	0 (0)	4 (40.0)
	Reptile	0 (0)	0 (0)	0 (0)
	Total	43	0	10
*Culex theileri*	Bird	2 (3.7)	3 (33.3)	0 (0)
	Human	9 (16.7)	3 (33.3)	0 (0)
	Non-human mammal	43 (79.6)	3 (33.3)	1 (100)
	Reptile	0 (0)	0 (0)	0 (0)
	Total	54	9	1

*Abbreviation*: *n* number of samplings

## Discussion

Field data on the host-feeding patterns provide important information to determine the potential vector capacity of hematophagous arthropods [7]. In the present study, 20 mosquito taxa were collected in Iran, which fed on nine mammal species, three bird species and one reptile taxon. The mosquito species had a significant overlap in their host-feeding patterns, i.e. most of the species shared one or more host species. As previously highlighted in similar studies from Switzerland, Germany and the USA [17, 27, 33], most of the analysed mosquito taxa fed on humans. In addition, only humans and cattle comprised nearly three-quarter of all analysed blood meals and 55% of all mosquito taxa fed on both host species. There are two potential explanations for the observed host similarities between the mosquito species: a strong overlap in the host preference or a relative high abundance of the selected hosts present in the available host communities. It can therefore be deduced that in comparison to the species-specific vector competence or host availability, species-specific host choices are probably not the most important factor driving pathogen transmission.

However, from an epidemiological point of view, feeding on multiple host species is more important to determine the vectors capacity for zoonotic pathogens. Exclusively based on the host-feeding patterns, most of the mosquito species must be considered as potential vectors of zoonotic pathogens [34]. The four most common mosquito species in this study (*Cx. pipiens pipiens* f. *pipiens*, *Cx. theileri*, *Cx. sitiens* and *Cx. perexiguus*) all fed on humans, non-human mammals and, to a certain extent, birds. Therefore, these species must be classified as potential vectors for pathogens transmitted between mammals (e.g. filarial nematodes [3]) and between birds and mammals (e.g. WNV [35] or SINV [36, 37]). Domestic mammals and particularly cattle were the second most commonly detected sources of blood meals after humans. This demonstrates the potential transmission risk of zoonotic pathogens by these four *Culex* species, e.g. cattle are potential reservoirs of the Rift Valley fever virus [38] or Batai virus [39, 40]. However, the host feeding patterns are only one part of vector capacity. The actual potential of a species as a vector has to be further tested using vector competence studies, i.e. to verify the ability of a mosquito to acquire a pathogen and subsequently transmit it to a new host.

The host-feeding preference of the most abundant species, *Cx. pipiens pipiens* f. *pipiens*, is commonly described as predominantly ornithophilic [41–44]. Although the species had the highest proportion of birds as source of blood meal in this study, there is increasing evidence from laboratory experiments [45] and field studies [17, 46] that the species regularly feeds on mammals. Indeed, in the present study, this species was found to blood-feed on humans and non-human mammals, as well as birds. One possible explanation for the reports of different host-feeding patterns of the species could be the composition of the host communities in the different study areas, e.g. *Cx. pipiens* (*s.l.*) feeding preference was found to be directly influenced by the change of host abundance during the course of the year, which also affected the epidemiology of pathogens [47, 48]. Due to the wide distribution and abundance of *Cx. pipiens pipiens* f. *pipiens* in Iran [3, 19], the species must be considered as both a potential enzootic and bridge vector for pathogens predominantly transmitted between birds [49, 50], e.g. SINV [1] or WNV [2] already circulating in the country. However, the same species is also a potential vector of pathogens, which can spill-over from non-human mammals to humans, e.g. filarial nematodes (*Dirofilaria immitis* or *Setaria labiatopapillosa*) also present in Iran [3].

This study gives a preliminary understanding of the vertebrate hosts of several mosquito species in Iran. Further studies should focus on an extended spatial-temporal sampling in other areas of the country, covering different biotopes to give a thorough understanding of the host-feeding patterns of the native mosquito fauna. In addition, as discussed in a previous publication [17], mosquitoes can have a high plasticity and host-feeding patterns therefore probably only reflect a high similarity in the host preference or availability of hosts [10]. Therefore, information on the local host communities is required to understand the spatial-temporal variability of host-feeding patterns, which was found to directly respond to the seasonality of the host abundance and thus also affecting pathogen epidemiology [48, 51]. Finally, the analyzed specimens originated from a study on the distribution of mosquito-borne pathogens not directly focused on systematically evaluating the host-feeding-patterns of mosquitoes [21]. The carbon dioxide baited traps used in this study target host-seeking females, but not blood-fed or engorged specimens. In addition, previous studies have already discussed the impact of different trapping methods on the collection of adult mosquitoes [52] and the analysis of blood-feeding patterns in general [17, 44]. Although statistically not significant, this study indicated an impact of the trapping method on the identified host-feeding patterns, e.g. human blood sources were more frequently caught with BG trap compared with the aspirator for *Cx. pipiens pipiens* f. *pipiens* and *Cx. perexiguus*. Our study did not apply the same trapping effort per trapping site. This probably prevents a comprehensive understanding of the observed host-feeding patterns. For example, the here presented study is significantly biased by an association between the detected host species and the trapping site (Additional file 7: Table S6). Therefore, standardizing data collection methods and sampling effort, with a variety of methods in each trapping site, would mean a more representative characterization of the hosts facilitated by different mosquito species could be acquired.

## Conclusions

In the Middle East including Iran, the analyses of host-feeding patterns of mosquitoes is a highly neglected field of research. The analyses of blood-fed Iranian mosquito species indicated a clear overlap and aggregation of host taxa, i.e. most mosquito species fed on different host groups (humans, mammals and birds) with the highest frequencies for humans and cattle. This information suggests that most species can potentially transmit pathogens between mammals as well as between mammals and birds, which might be the result of a similar host selection or a high dependence on the host availability. In conclusion, pathogen transmission cycles are probably significantly influenced by the local composition of the host communities. Furthermore, contrary to the frequently published opinion, *Cx. pipiens pipiens* f. *pipiens* was not predominantly ornithophilic, but fed on a diverse range of vertebrate hosts including humans, non-human mammals, birds and even reptiles, highlighting the species’ role as a potential enzootic and bridge vector. Therefore, the breeding sites of this species (e.g. artificial water bodies) should be included in control measurements for currently circulating pathogens in Iran. In addition, the present study also demonstrates the relevance of studies on the host-feeding patterns of mosquitoes to understand one of the most important parts of species vector capacity and pathogen transmission cycles.

## Additional files

Additional file 1: **Table S1.** Blood-fed mosquitoes collected in Iran in the years 2015 and 2016 with information on the coordinates of the trapping site, elevation, biome, land use, trapping method, trapping date, mosquito species and identified source of blood meal. (XLSX 31 kb)

Additional file 2: **Table S2.** Frequency of collected mosquito specimens with information on the frequency/percentage of blood-fed mosquito specimens per trapping site, method and date. (XLSX 18 kb)

Additional file 3: **Table S3.** Frequency/percentage of mosquito specimens per trapping method. (XLSX 11 kb)

Additional file 4: **Table S4.** Frequency of blood meals per host species taken by the least abundant mosquito species. (XLSX 11 kb)

Additional file 5: **Figure S1.** Frequency of blood meals on different hosts taken by the least abundant mosquito species differentiated for the trapping month, summed over the two sampling years (2015–2016). (JPG 604 kb)

Additional file 6: **Table S5.** Frequency for each host-feeding group (birds, non-human mammals, humans and reptiles) for the mosquito species not belonging to the four most abundant blood-fed mosquito species differentiated for the three used trapping methods. (XLSX 10 kb)

Additional file 7: **Table S6.** Frequency/percentage for the host-feeding groups (birds, non-human mammals, humans and reptiles) for each trapping site. (XLSX 15 kb)

## Abbreviations

AIC: Akaike information criterion
BG trap: Biogents Sentinel trap
cf: Confer
ELISA: Enzyme-linked immunosorbent assay
EVS trap: Heavy Duty Encephalitis Vector Survey trap
SINV: Sindbis virus
WNV: West Nile virus
PCR: Polymerase chain reaction
