# Supplementary figures and images for: Host-feeding patterns of Culex mosquitoes in Iran
Source: Parasit Vectors. 2018 Dec 27;11:669. doi: 10.1186/s13071-018-3237-2 (PMC6307250; doi:10.1186/s13071-018-3237-2)

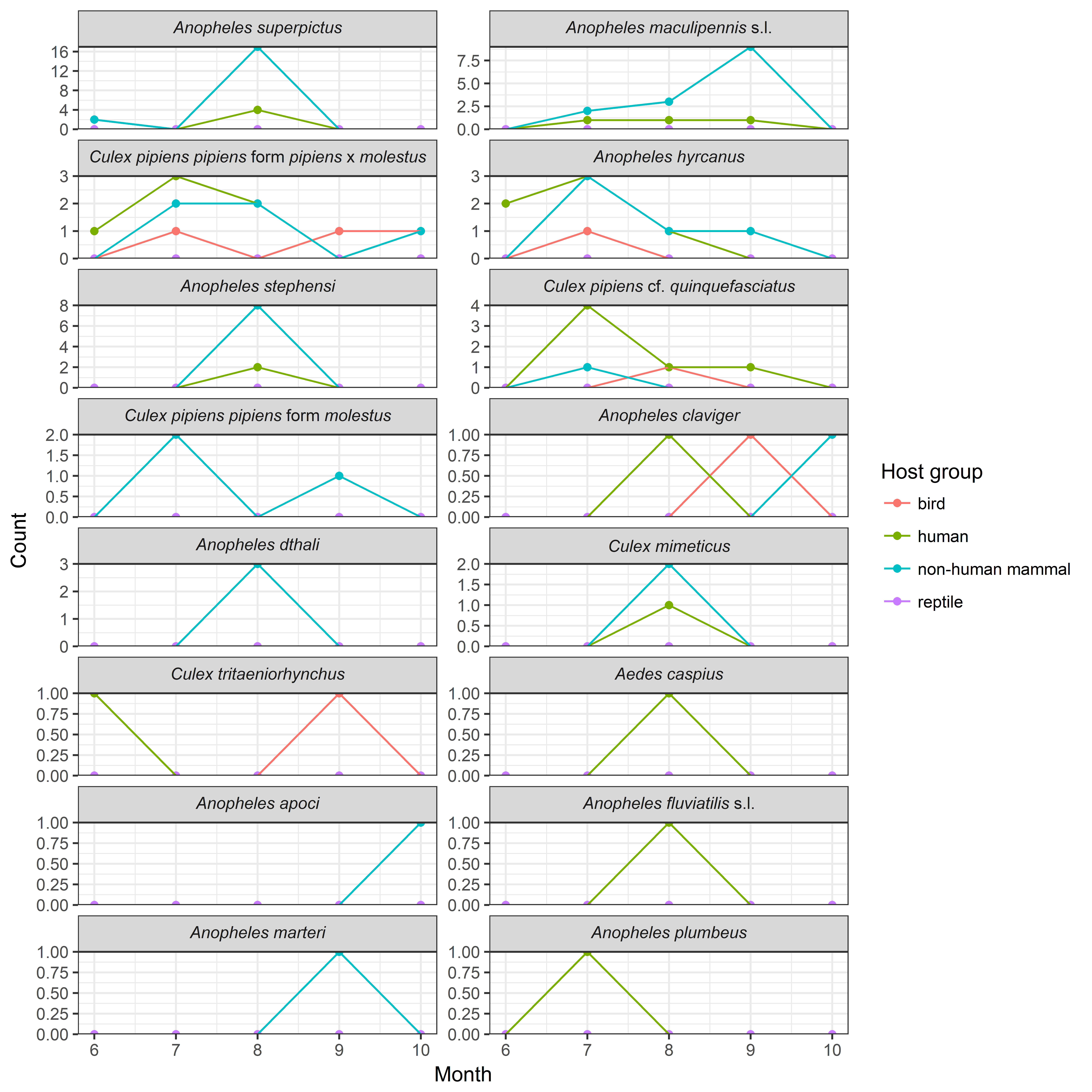

Supplement: Supplementary file 5 — Figure S1. Frequency of blood meals on different hosts taken by the least abundant mosquito species differentiated for the trapping month, summed over the two sampling years (2015–2016). (JPG 604 kb) [file 13071_2018_3237_MOESM5_ESM.jpg]
